# Medical Association of Missouri

**Published:** 1883-02-20

**Authors:** 


### *MEDICAL ASSOCIATION OF MISSOURI.*

Transactions of the Medical Association of the State of Missouri, at its 28th Annual session held at Hannibal, May 16, 1882. A neatly gotten up volume of 220 pages.

The papers read were able and interesting. The address of Dr. W. P. King, the president, on the subject of Quacks and Quackery, is of special interest, and must have furnished a rare treat to the members of the Association. It shows up Missouri in rather a bad light in the great number of Quacks with whom she is infested. There are other papers of interest, on the following subjects:

Phlegmasia Alba Dolens—*Schenck.* Railroad Surgery—*Trader.* Hot Water Injections in Uterine Disease—*Schenck.* Diseases of Children—*Kingsley.* Cystic Degeneration of the Thyroid Gland—*McAlester.* Pott's Disease in the Upper Spine—*Steele.* Congenital Cysts of the Thyroid Gland—*Lutz.* Neuratrophia—*Hughes.* Hydrophobia—*Matthews.* Idiopathic Sub-Acute Laryngitis—*Glasgow.* Mortality among Puerperal Women—*Hurt.* Vaccination and Inoculation—*Brooks.* Quinine: Its Use and Abuse—*Dewey.* Placenta Previa—*Warth.* Meningocele—*Warth.* Local Medical Organizations for Missouri—*Norris.* State Medicine and Medical Legislation—*Special Committee.*

OFFICERS ELECT FOR 1882–'83.

President—A. E. GORE, Paris.

Vice-Presidents—Pinckney French, Mexico; R. F. Brooks, Carthage; P. S. Fulkerson, Lexington; O. D. Fitzgerald, Lathrop; F. J. Lutz, St. Louis.

Recording Secretaries—C. A. Todd, St. Louis; J. H. Duncan, Columbia. Corresponding Secretary—Wm. Dickinson, St. Louis.

Treasurer—C. A. Thompson, Jefferson City.

The next Annual meeting will be held at Jefferson City, Cole County, Missouri, Tuesday, Wednesday and Thursday, May 15, 16 and 17, 1883.

